# Severity and 28-day in-hospital mortality of interleukin-6, neutrophil to lymphocyte ratio, and APACHE II score in patients with sepsis

**DOI:** 10.3389/fcimb.2026.1690126

**Published:** 2026-03-25

**Authors:** Sheng Yue, Xiaojing Du, Xiaohui Li, Lingshan Zhou

**Affiliations:** General Intensive Care Unit, Fuwai Central China Cardiovascular Hospital, Henan Provincial People’s Hospital, Zhengzhou, Henan, China

**Keywords:** 28-day in-hospital mortality, APACHE II score, IL-6, NLR, sepsis, septic shock, severity

## Abstract

**Objective:**

This paper investigated the predictive value of interleukin-6 (IL-6), neutrophil-to-lymphocyte ratio (NLR), and APACHE II score for 28-day in-hospital mortality in septic patients.

**Methods:**

A total of 153 septic patients were retrospectively included, and their general clinical data and laboratory indices were collected, followed by counting 28-day in-hospital survival. Patients were allocated into sepsis and septic shock groups following the disease severity and death group and survival group according to their 28-day in-hospital survival. The predictive value of IL-6, NLR, and APACHE II scores for septic shock and 28-day in-hospital mortality and risk factors impacting 28-day in-hospital survival in septic patients were examined.

**Results:**

Serum IL-6, NLR values, and APACHE II scores were significantly higher in patients with septic shock and in those with 28-day in-hospital mortality (*P* < 0.05). The combined detection of IL-6, NLR, and APACHE II score had higher predictive value for septic shock and 28-day in-hospital mortality compared to any single indicator (combined AUC: 0.873/0.938; individual AUCs: 0.731/0.764, 0.783/0.820, 0.776/0.812), and their combination demonstrated increased sensitivity and specificity (all above 75%). IL-6, NLR, and APACHE II score were independent risk factors for septic shock and 28-day in-hospital mortality (*P* < 0.05). Elevated IL-6, NLR, and APACHE II scores contributed to increased risk of 28-day in-hospital mortality in septic patients (*P* < 0.05).

**Conclusion:**

IL-6, NLR, and APACHE II score have predictive value and are independent risk factors for septic shock and 28-day in-hospital mortality.

## Introduction

Sepsis is defined as a systemic inflammatory response syndrome (SIRS) caused by pathogenic microorganisms such as bacteria, fungi, or viruses, manifested with an uncontrolled inflammatory response and impaired multiple organ function, which progresses rapidly, further inducing septic shock (Mushtaq and Kazi, 2022). Sepsis is a crucial public health problem worldwide, which mainly occurs in intensive care unit (ICU) patients, with increased incidence and mortality (Evans, 2018; Prescott and Angus, 2018). Management of septic patients mainly involves controlling underlying infection, stabilizing hemodynamics, and regulating the host response (Vincent, 2022). Despite the continuous advancement of modern medicine, there are still many patients who die of sepsis each year (Fleischmann et al., 2016; Bauer et al., 2020). At present, the acute physiology and chronic health evaluation II (APACHE II) is often applied in the severity evaluation of sepsis and septic shock patients, but the evaluation process is cumbersome, and there is a lack of effective serological indicators for severity and prognostic assessment (Coopersmith et al., 2018; Barichello et al., 2022; Yuan et al., 2024). The 2021 guidelines put forward the urgent need to improve the diagnostic accuracy, modify the therapeutic methods, and increase the survival rate. Early detection and timely management of biomarkers that can predict the mortality of patients with sepsis are of crucial importance to improve its prognosis (Evans et al., 2021; Prescott and Ostermann, 2023).

Interleukin-6 (IL-6), as a pro-inflammatory cytokine generated by T lymphocytes, fibroblasts, endothelial cells, and monocytes, shows a pathological impact on chronic inflammation as well as autoimmunity (Tanaka et al., 2014; Jones et al., 2018; Alter et al., 2023). IL-6 is linked with the risk of shock and death in septic patients (Palalioglu et al., 2024), and many inflammatory mediators in sepsis can stimulate the production of IL-6 (Holmes et al., 2003). However, the value of IL-6 in terms of diagnosis and prognosis remains controversial. Chan et al. found that IL-6 served as a useful biomarker for diagnosing sepsis and prognosis assessment. Serum IL-6 levels significantly increased in the course of sepsis, which were higher during the progression to septic shock or even death (Feng et al., 2016). However, a previous meta-analysis on the diagnostic performance of IL-6 showed only a moderate success rate in distinguishing adult sepsis from non-infectious SIRS (Ma et al., 2016). Therefore, it is necessary to combine it with other biomarkers as a diagnostic strategy for sepsis. Lymphocytes are important immune cells implicated in the occurrence and development of sepsis, and cell counts such as neutrophil-to-lymphocyte ratio (NLR) can serve as an independent prognostic factor for sepsis (Kumar, 2018; Zhang et al., 2024). NLR, a systemic inflammatory indicator based on white blood cell count (WBC), increases with the progression of inflammatory diseases (Huang et al., 2020). Currently, there is scarce research on the impact of serum IL-6 and NLR, as well as APACHE II scores, on the severity and prognosis of septic patients. This study focused on the evaluation value of IL-6, NLR, and the APACHE II score on disease severity and prognosis to seek new diagnostic and prognostic approaches for septic patients.

## Materials and methods

### Ethical approval

The studies involving humans were approved by Ethics Committee of Fuwai Central China Cardiovascular Hospital, Henan Provincial People’s Hospital. The studies were conducted in accordance with the local legislation and institutional requirements. The ethics committee/institutional review board waived the requirement of written informed consent for participation from the participants or the participants’ legal guardians/next of kin because the retrospective nature of this study and the use of de-identified data.

### Participants

In this paper, 184 patients with sepsis admitted to Fuwai Central China Cardiovascular Hospital and Henan Provincial People’s Hospital from October 2021 to February 2024 were retrospectively selected, of which 19 cases were not enrolled due to withdrawal of treatment or incomplete data, 12 cases were excluded following the inclusion and exclusion criteria, and 153 patients with sepsis were ultimately enrolled. These eligible patients were classified into sepsis (SP, *N* = 97) and septic shock (SPS, *N* = 56) groups according to the disease severity, and the sepsis patients were assigned into the death (*N* = 58) and the survival (*N* = 95) groups based upon whether or not they had died within 28 days in the hospital.

### Inclusion and exclusion criteria

Inclusion criteria: (1) the patients conformed to the diagnostic criteria for sepsis or septic shock in the Third International Consensus Definitions for Sepsis and Septic Shock (Sepsis-3) (SSC 2016) (Shankar-Hari et al., 2016), (2) patients aged 18–80 years old, (3) the time between the onset of the disease and the patient’s admission to the ICU < 24h, and (4) patients did not receive relevant treatment before admission.

Exclusion criteria: (1) those with a history of severe heart failure, chronic liver failure, or chronic kidney disease; (2) those complicated with malignancies or venous thromboembolism; (3) those on long-term use of corticosteroids or immunosuppressants; (4) those complicated with severe acute central nervous system diseases, including trauma, ischemic or hemorrhagic encephalopathy; (5) pregnant and lactating women; (6) those who withdrew from treatment or who had incomplete medical records.

Diagnostic criteria for sepsis: a Sepsis-related Organ Failure Assessment (SOFA) score of ≥ 2 points.

Diagnostic criteria for septic shock: met sepsis’s diagnostic criteria and still required pressor medication to keep mean arterial pressure ≥ 65 mmHg or higher with lactate > 2 mmol/L despite aggressive fluid resuscitation.

### Collection of medical records

Clinical information within 24h of admission and biochemical indicators that may impact the prognosis of all patients were harvested via the electronic medical record system, consisting of gender, age, comorbidities (diabetes mellitus, coronary artery disease, and hypertension), site of infection, days on ventilator, and length of stay in the ICU, and the prognosis of the patients was counted for the first 28 days post-admission to the ICU.

An APACHE II score (Godinjak et al., 2016) was acquired at the time of admission, and it consisted of the evaluation items of the patient’s age, chronic health condition, and acute pathophysiological changes, with a score of 0 to 71, which comprehensively reflected the severity of the patient’s condition, with a higher score representing a more serious condition.

Other laboratory indicators upon admission were collected, including platelet count (PLT), prothrombin time (PT), activated partial thromboplastin time (APTT), IL-6, neutrophil count (NEU), lymphocyte count (LYM), NLR, WBC, procalcitonin (PCT), C-reactive protein (CRP), lactic acid (Lac), as well as serum creatinine (Scr). All laboratory indicators were acquired from a single blood sample obtained within 24h of admission.

### Statistical methods

Data analysis and graphing were implemented with SPSS statistical software (27.0, SPSS, Inc., Chicago, IL, USA) combined with GraphPad Prism software (8.0, GraphPad Software Inc., San Diego, CA, USA). The normal distribution of data was examined with the Kolmogorov–Smirnov test; measurement data in normal distribution, indicated as mean ± standard deviation, were processed with the independent samples *t*-test for two-group comparisons; measurement data that did not obey normal distribution were expressed as median (interquartile range) [M (IQR)], with the Mann-Whitney *U* test for two-group comparisons. Categorical data were indicated as numbers and percentages, with a chi-square test for two-group comparisons. ROC curves were adopted to evaluate the predictive value of IL-6, NLR, and APACHE II scores alone and in combination on the severity and prognosis in septic patients. MedCalc software (20.0.15, from MedCalc Software Ltd., Ostend, Belgium) was adopted for comparing differences in area under the ROC curve (AUC). Survival analysis was implemented with the Kaplan–Meier method, and survival curves were plotted and analyzed with the log-rank test. Variables with *P* < 0.05 in the univariate analysis were recruited in the multivariate logistic regression analysis, and the variables were entered using the Enter method, and the odds ratio (OR) values and their 95% confidence intervals (CIs) were calculated. Statistical significance was reflected in a value of *P* < 0.05.

## Results

### Comparison of general clinical baseline data

Comparative analysis of the clinical baseline data of the two groups revealed no differences between septic shock patients and septic patients in terms of age, gender, site of infection, comorbidities, days on ventilator, length of stay in the ICU, APTT, PLT, LYM, and Scr (all *P* > 0.05), while noticeable differences were witnessed in PT, IL-6, WBC, NEU, NLR, PCT, Lac, CRP, and APACHE II scores (all *P* < 0.05). Subsequently, all subjects were further classified into survival (*N* = 95) and death (*N* = 58) groups based upon whether or not they had died within 28 days in the hospital. No differences were found between patients in the survival and the death groups in terms of age, gender, site of infection, comorbidities, days on ventilator, length of stay in the ICU, PT, APTT, LYM, and Scr (all *P* > 0.05), with remarkable differences in terms of IL-6, PLT, WBC, NEU, NLR, PCT, Lac, CRP, and APACHE II scores (all *P* < 0.05) (**Table 1**).

**Table 1:** Comparison of clinical baseline data between two groups.

Clinical indicator	Sepsis group (N = 97)	Septic shock group (N = 56)	*Pa* value	Survival group (N = 95)	Death group (N = 58)	*Pb* value
Age (years)	60.23 ± 10.55	63.04 ± 12.24	0.137	59.89 ± 10.73	63.48 ± 11.79	0.055
Gender (male/female, case)	56/41	25/31	0.178	56/39	25/33	0.067
Site of infection (n [%])						
Respiratory system	39 (40.21)	25 (44.64)	0.613	39 (41.05)	25 (35.21)	0.519
Digestive system	23 (23.71)	9 (16.07)	0.307	21 (22.11)	11 (18.97)	0.687
Urinary system	18 (18.56)	17 (30.36)	0.111	19 (20.00)	16 (27.59)	0.2323
Comorbidities (n [%])						
Hypertension	50 (51.55)	25 (44.64)	0.502	48 (50.53)	27 (46.55)	0.739
Diabetes mellitus	17 (17.53)	12 (21.43)	0.669	16 (16.84)	13 (22.41)	0.403
Coronary artery disease	9 (9.38)	11 (19.64)	0.083	9 (9.47)	11 (18.97)	0.136
Days on ventilator (day)	7 (4,9)	7 (6,9)	0.109	7 (4,9)	7 (6.9)	0.061
Length of stay in the ICU (day)	14.15 ± 5.39	15.77 ± 4.32	0.058	14.13 ± 5.53	15.76 ± 4.05	0.053
PT (s)	14.76 ± 3.05	15.89 ± 3.09	0.029	14.79 ± 3.05	15.80 ± 3.12	0.052
APTT (s)	42.48 ± 5.10	44.08 ± 4.36	0.051	42.45 ± 5.32	44.01 ± 4.06	0.058
IL-6 (ng/L)	34.54 ± 11.00	43.39 ± 10.80	0.001	33.18 ± 10.84	45.31 ± 8.85	0.001
PLT (× 10^9^/L)	163.40 (132.10, 190.40)	177.20 (136.20, 199.40)	0.103	160.30 (102.30, 188.50)	179.00 (138.00, 201.80)	0.028
WBC (× 10^9^/L)	15.59 ± 5.57	17.69 ± 7.36	0.048	15.54 ± 5.64	17.47 ± 7.21	0.041
NEU (× 10^9^/L)	9.25 (6.72,12.10)	12.50 ± 5.80	0.001	9.21 (6.65, 12.50)	11.07 (8.15, 15.56)	0.004
LYM (× 10^9^/L)	1.01 (0.80, 1.27)	0.98 (0.75, 1.06)	0.051	1.03 (0.80, 1.28)	0.98 (0.75, 1.07)	0.065
NLR	9.54 (7.49, 11.67)	12.97 (10.24, 15.28)	0.001	9.55 (7.58, 11.63)	12.64 (10.03, 15.58)	0.001
PCT (ng/mL)	2.17 (1.90, 2.43)	2.47 (1.79, 3.79)	0.024	2.17 (1.89, 2.42)	2.62 (1.83, 3.79)	0.003
Lac (mmol/L)	7.44 ± 3.35	10.23 ± 4.00	0.001	7.23 ± 3.30	10.48 ± 3.83	0.001
CRP (mg/L)	99.65 (71.47, 128.70)	119.00 (79.03, 158.00)	0.047	97.44 (70.36, 127.50)	116.80 (79.10, 158.30)	0.036
Scr (μmol/L)	146.50 ± 38.77	155.00 ± 53.02	0.063	146.30 ± 38.84	160.30 ± 52.48	0.060
APACHE II score	15 (12, 18)	20 (17, 25)	0.001	15 (12, 18)	21 (17, 25)	0.001

PT, prothrombin time; APTT, activated partial thromboplastin time; PLT, platelet count; IL-6, interleukin 6; WBC, white blood cell; NEU, neutrophil count; LYM, lymphocyte count; NLR, neutrophil to lymphocyte ratio; PCT, procalcitonin; Lac, lactic acid; CRP, C-reactive protein; Scr, serum creatinine. Measurement data in normal distribution were presented as mean ± standard deviation, with independent samples t-test for two-group comparisons; measurement data did not obey normal distribution were expressed as M (IQR), with Mann-Whitney U test for two-group comparisons. Categorical variables were comparatively analyzed using Fisher's exact test. Statistical significance was reflected in a value of *P* < 0.05.

### Diagnostic value of IL-6, NLR, and APACHE II score for septic shock

**Figure 1:**
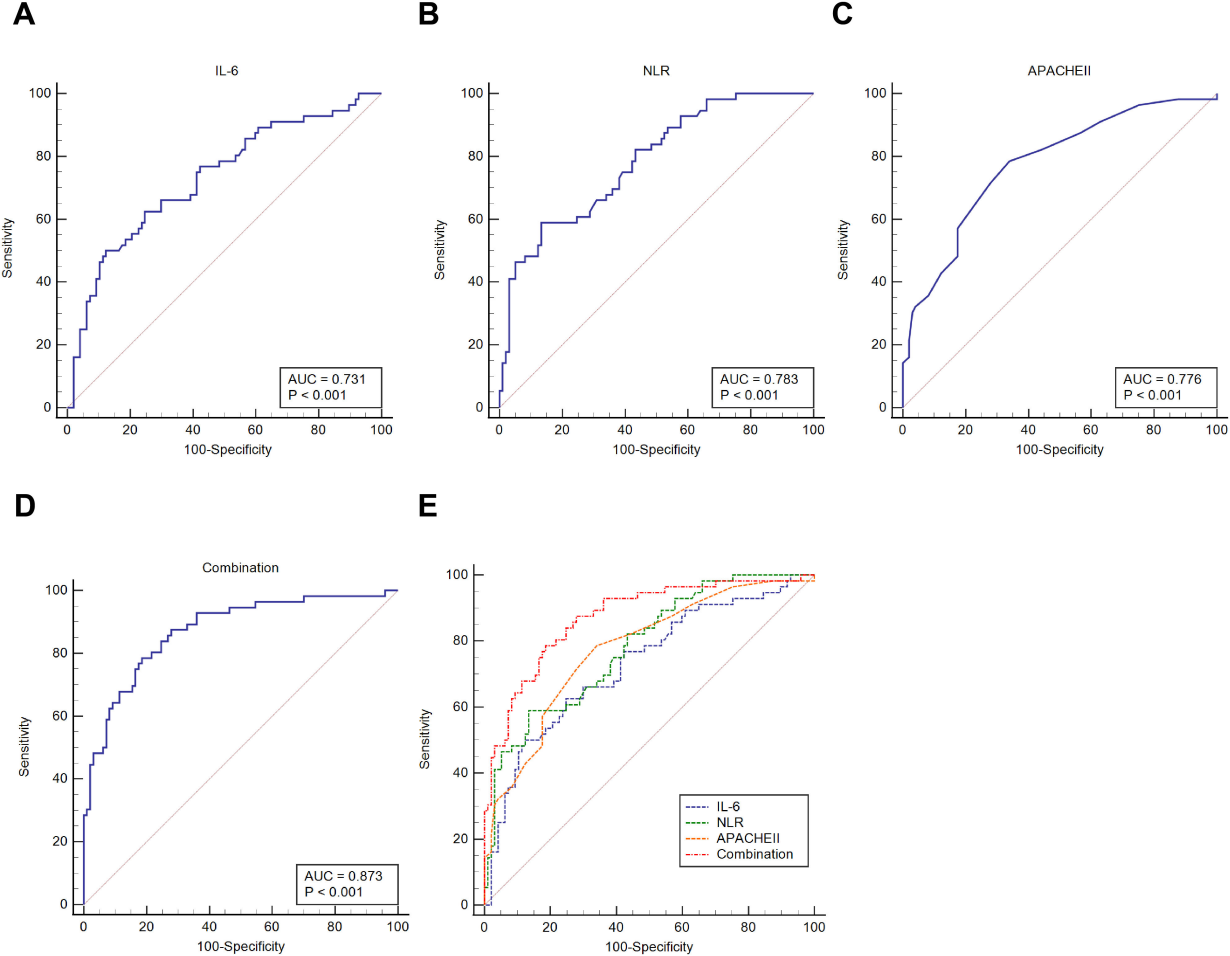
ROC curve for analyzing the predictive value of IL-6, NLR, and APACHE II score on septic shock. **(A–D)**. ROC curve analysis to assess the predictive value of serum IL-6, NLR, and APACHE II scores and the combination of these three indicators on septic shock; **(E)**. MedCalc analysis of the difference in the area under the ROC curve of these three indicators on the occurrence of septic shock.

The ROC curve was subsequently plotted to disclose the clinical diagnostic value of serum IL-6, NLR, and APACHE II score on the severity of septic patients, which showed that the AUC of serum IL-6 for diagnosing septic shock was 0.731 (cutoff value = 41.97 mg/L, sensitivity = 62.50%, and specificity = 75.26%); the AUC of NLR for diagnosing septic shock was 0.783 (cutoff value = 12.36, sensitivity = 58.93%, and specificity = 86.60%); and the AUC of APACHE II score for diagnosing septic shock was 0.776 (cutoff value = 16, sensitivity = 78.57%, and specificity = 65.98%). In addition, the AUC of IL-6 and NLR combined with the APACHE II score for diagnosing septic shock was 0.873 (cutoff value = 0.391, sensitivity = 78.57%, and specificity = 81.44%). The diagnostic value of the combined test of the three parameters for the severity of sepsis patients was higher than the diagnostic value of IL-6, NLR, and APACHE II score alone (*P* < 0.05) (**Table 2**; **Figures 1A–E**).

**Table 2:** Pairwise comparison of ROC curves for IL-6, NLR, APACHE II scores and their combination.

Item	AUC	95%CI	Sensitivity	Specificity
IL-6	0.731	0.653 – 0.799	62.50%	75.26%
NLR	0.783	0.710 – 0.846	58.93%	86.60%
APACHE II score	0.776	0.702 – 0.840	78.57%	65.98%
Combination	0.873	0.809 – 0.921	75.57%	81.44%
IL-6~Combination	*P* = 0.0002			
NLR~Combination	*P* = 0.0046			
APACHE II score~Combination	*P* = 0.0022			

Multiple ROC area under the curve (AUC) comparisons were performed using the Delong test in MEDCALC software. ROC curve, receiver operating characteristic curve; IL-6, interleukin-6; NLR, neutrophil to lymphocyte ratio; APACHE II, Acute Physiology and Chronic Health Evaluation II.

### Serum IL-6, NLR, and APACHE II score are independent risk factors for sepsis severity

Further using septic shock as the dependent variable (sepsis = 0, septic shock = 1), the PT, IL-6, WBC, NEU, NLR, PCT, Lac, CRP, and APACHE II scores were included as independent variables in **Table 1** with *P*a < 0.05 in the univariate logistic regression analysis. The findings suggested that the PT, IL-6, NEU, NLR, PCT, Lac, CRP, and APACHE II score were all independent risk factors in the occurrence of shock in septic patients (all *P* < 0.05). Subsequently, PT, IL-6, NEU, NLR, PCT, Lac, CRP, and APACHE II scores were recruited in the multivariate logistic regression analysis, which revealed that IL-6 (*P* = 0.041, OR = 1.053), NLR (*P* = 0.001, OR = 1.638), and APACHE II score (*P* = 0.002, OR = 1.191) were independent risk factors for the development of shock in septic patients, as shown in **Table 3**.

**Table 3:** Logistic regression analysis of independent risk factors for the degree of condition in patients with sepsis.

Clinical indicators	Univariate logistic regression analysis			Multivariate logistic regression analysis		
	*P* value	*OR* value	*95% CI*	*P* value	*OR* value	*95% CI*
PT	0.031	1.128	1.011 – 1.259	0.516	0.928	0.740 – 1.163
IL-6	0.001	1.078	1.042 – 1.117	0.041	1.053	1.002 – 1.106
WBC	0.051	1.054	1.000 – 1.1111	–	–	–
NEU	0.001	1.144	1.057 – 1.237	0.085	0.877	0.755 – 1.018
NLR	0.001	1.422	1.246 – 1.622	0.001	1.638	1.303 – 2.061
PCT	0.001	1.956	1.329 – 2.879	0.311	1.327	0.768 – 2.294
Lac	0.001	1.233	1.116 – 1.363	0.097	1.128	0.978 – 1.301
CRP	0.015	1.010	1.002 – 1.018	0.684	0.996	0.979 – 1.014
APACHE II score	0.001	1.228	1.134 – 1.329	0.002	1.191	1.068 – 1.328

PT, prothrombin time; PLT, platelet count; IL-6, interleukin 6; WBC, white blood cell; NEU, neutrophil count; LYM, lymphocyte count; NLR, neutrophil to lymphocyte ratio; PCT, procalcitonin; Lac, lactic acid; CRP, C-reactive protein; APACHE II, Acute Physiology and Chronic Health Evaluation II.

### Predictive value of IL-6, NLR, and APACHE II score for 28-day in-hospital mortality in sepsis

**Figure 2:**
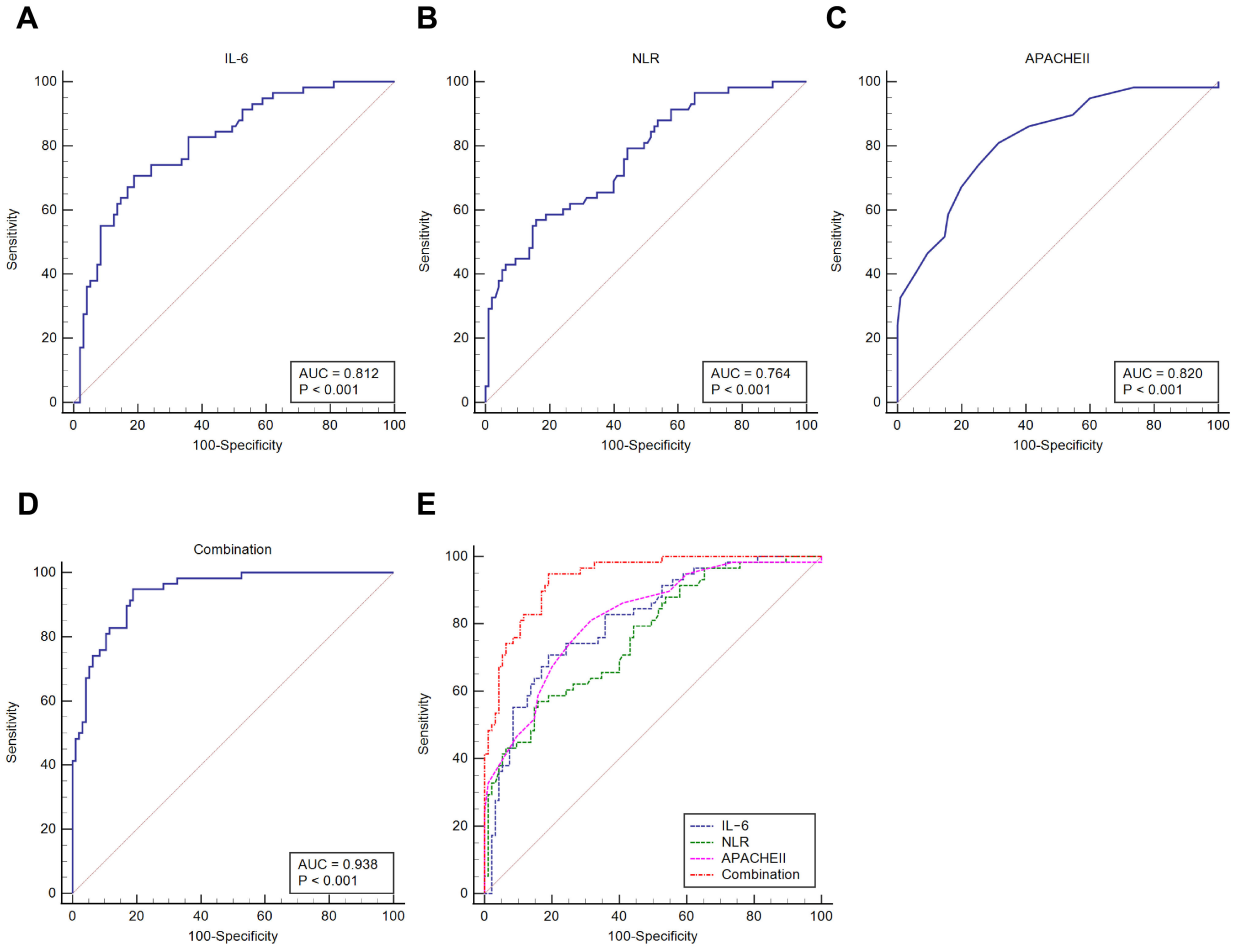
ROC curve for analyzing the predictive value of IL-6, NLR, and APACHE II score for 28-day in-hospital mortality in sepsis. **(A–D)**. ROC curve analysis to evaluate the predictive value of serum IL-6, NLR, and APACHE II scores and the combination of these three indicators on sepsis prognosis; **(E)**. MedCalc analysis of the difference in the area under the ROC curve of these three indicators on 28-day in-hospital mortality of sepsis.

IL-6, NLR, and APACHE II scores were utilized as screening indices to predict the 28-day in-hospital mortality of patients with sepsis. ROC curve analysis unveiled that the AUC of serum IL-6 value to diagnose 28-day in-hospital mortality of sepsis was 0.812 (optimal cutoff = 41.97 mg/L, sensitivity = 70.69%, and specificity = 81.05%); the AUC of NLR value was 0.764 (optimal cutoff = 12.23, sensitivity = 56.90%, and specificity = 84.21%); and the AUC for the APACHE II score was 0.820 (optimal cutoff = 16.00, sensitivity = 81.03%, and specificity = 68.42%). The AUC for IL-6 and NLR combined with the APACHE II score for prediction of 28-day in-hospital mortality in sepsis was 0.941 (optimal cutoff = 0.248, sensitivity = 94.83%, and specificity = 82.11%). Besides, MedCalc analysis of the difference in the AUC indicated that the predictive efficacy of the combination of all three indices for 28-day in-hospital mortality in septic patients was higher than the predictive efficacy of IL-6, NLR, and APACHE II score alone (*P* < 0.05). It is suggested that IL-6 and NLR combined with the APACHE II score, are indicators for diagnosing 28-day in-hospital mortality of septic patients, and the combination of all three indices has a higher predictive value for 28-day in-hospital mortality of septic patients (**Table 4**; **Figures 2A–E**).

**Table 4:** Pairwise comparison of ROC curves for IL-6, NLR, APACHE II scores and their combination.

Item	AUC	95%CI	Sensitivity	Specificity
IL-6	0.812	0.741 – 0.870	70.69%	81.05%
NLR	0.764	0.689 – 0.829	56.90%	84.21%
APACHE II score	0.820	0.749 – 0.877	81.03%	68.42%
Combination	0.938	0.888 – 0.971	94.83%	81.05%
IL-6~Combination	*P* = 0.0002			
NLR~Combination	*P* < 0.0001			
APACHE II score~Combination	*P* = 0.0001			

Multiple ROC area under the curve (AUC) comparisons were performed using the Delong test in MEDCALC software.ROC curve, receiver operating characteristic curve; IL-6, interleukin-6; NLR, neutrophil to lymphocyte ratio; APACHE II, Acute Physiology and Chronic Health Evaluation II.

### Serum IL-6, NLR levels, and APACHE II score are independent risk factors for 28-day in-hospital mortality in sepsis

Further using the 28-day in-hospital survival of septic patients as the dependent variable (survival = 0, death = 1), the days on ventilator, IL-6, PLT, WBC, NEU, NLR, PCT, Lac, CRP, and APACHE II scores with *P*b < 0.05 were recruited as independent variables in **Table 1**, and the findings demonstrated that IL-6, PLT, NEU, NLR, PCT, Lac, CRP, and APACHE II scores were all influencing factors for 28-day in-hospital mortality in septic patients (all *P* < 0.05). Subsequently, IL-6, PLT, NEU, NLR, PCT, Lac, CRP, and APACHE II scores were recruited in the multivariate logistic regression analysis, with the findings demonstrating that IL-6 (*P* = 0.001, OR = 1.202), NLR (*P* = 0.001, OR = 2.239), and APACHE II score (*P* = 0.001, OR = 1.401) were independent risk factors for 28-day in-hospital mortality in sepsis (**Table 5**).

**Table 5:** Logistic regression analysis of independent risk factors for 28-day in-hospital mortality in patients with sepsis.

Clinical indicators	Univariate logistic regression analysis			Multivariate logistic regression analysis		
	*P* value	*OR* value	*95% CI*	*P* value	*OR* value	*95% CI*
IL-6	0.001	1.128	1.081 – 1.178	0.001	1.202	1.100 – 1.314
PLT	0.016	1.009	1.002 – 1.017	0.112	1.013	0.997 – 1.030
WBC	0.043	1.056	1.002 – 1.113	0.165	0.867	0.709 – 1.060
NEU	0.001	1.156	1.067 – 1.252	0.066	0.810	0.647 – 1.014
NLR	0.001	1.386	1.222 – 1.572	0.001	2.239	1.525 – 3.287
PCT	0.001	2.332	1.521 – 3.576	0.052	2.406	0.993 – 5.835
Lac	0.001	1.292	1.161 – 1.438	0.085	1.190	0.976 – 1.451
CRP	0.011	1.010	1.002 – 1.019	0.974	1.000	0.971 – 1.031
APACHE II score	0.001	1.310	1.193 – 1.438	0.001	1.401	1.174 – 1.671

PLT, platelet count; IL-6, interleukin 6; WBC, white blood cell; NEU, neutrophil count; LYM, lymphocyte count; NLR, neutrophil to lymphocyte ratio; PCT, procalcitonin; Lac, lactic acid; CRP, C-reactive protein; APACHE II, Acute Physiology and Chronic Health Evaluation II.

### Elevated IL-6, NLR, and APACHE II scores significantly increase the risk of 28-day in-hospital mortality in sepsis

**Figure 3:**
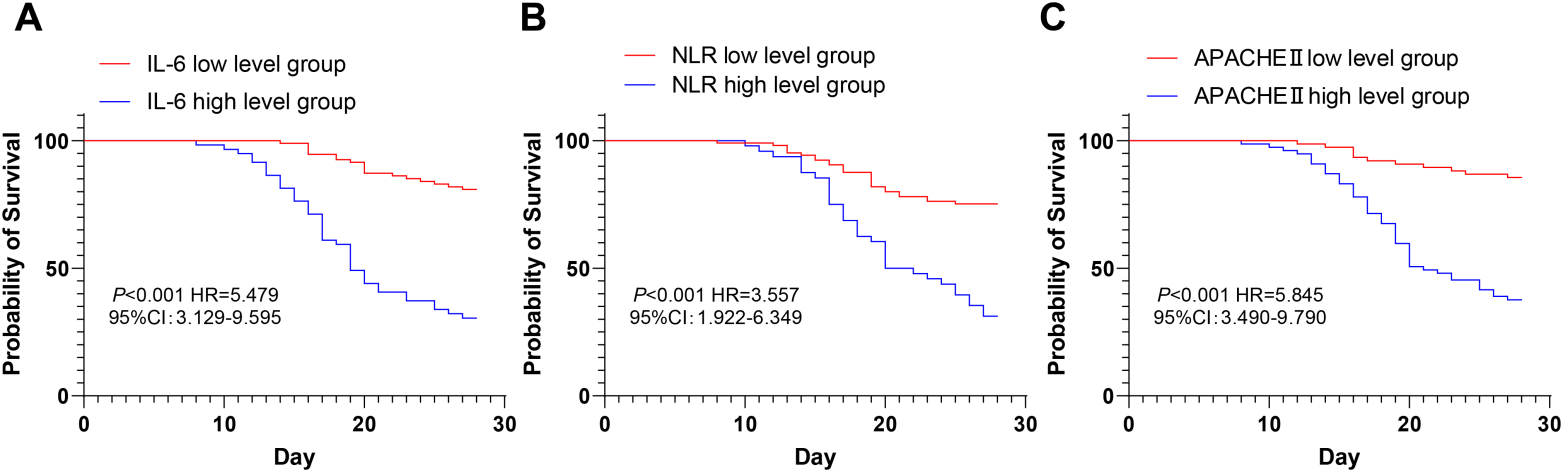
Kaplan–Meier survival curves for 28-day in-hospital mortality in septic patients. Kaplan–Meier curves were used to analyze the effects of IL-6 **(A)**, NLR **(B)**, and APACHE II score **(C)** on 28-day in-hospital survival in patients with sepsis.

According to the cutoff value of ROC curve analysis, septic patients were allocated into the IL-6 high/low level group, the NLR high/low level group, and the APACHE II high/low score group, and the effects of IL-6, NLR, and APACHE II scores on the 28-day in-hospital survival of septic patients were analyzed by Kaplan–Meier survival curves. It was revealed that 28-day in-hospital survival of patients with high IL-6, NLR, and APACHE II scores all decreased significantly over time, and elevated levels of IL-6, NLR, and APACHE II scores notably advanced the risk of 28-day in-hospital mortality in sepsis (**Figure 3**).

## Discussion

Dynamic changes in IL-6 levels reflect inflammatory intensity and possess moderate-to-good discriminatory ability for 28-day mortality (Varga et al., 2025). Similarly, a persistently elevated NLR is a commonly used indicator of systemic immune dysregulation and poor prognosis (Stoian et al., 2025). Previous efforts to combine IL-6 and NLR have shown improved specificity over single parameters (Liu et al., 2021). However, the synergistic integration of these biological markers with the classic APACHE II clinical scoring system remains under-explored. Additionally, the APACHE II score is a classic clinical tool for evaluating disease severity (Li et al., 2025), and higher APACHE II scores and IL-6 levels are related to an increased probability of death in septic patients (Li et al., 2023). The novelty of this study lies primarily in the following aspects: (1) This study for the first time combined IL-6, NLR, and APACHE II scores, effectively bridging the gap between molecular biomarkers and physiological scoring systems; (2) The combined model significantly outperformed individual parameters, achieving a robust balance of sensitivity and specificity (both >75%), which is critical for minimizing clinical misdiagnosis; (3) Elevated levels of IL-6, NLR, and APACHE II score increased in-hospital 28-day mortality risk in septic patients.

The dynamics of biomarkers in sepsis reflect a complex interplay between host-related factors (age, biological sex, comorbidities, and genetics), the specific pathogen, and the severity of multi-organ failure (Wang and Liu, 2023). Our findings indicated elevated levels of IL-6, WBC, NEU, NLR, PCT, Lac, CRP, and APACHE II scores in patients with septic shock and 28-day in-hospital mortality. However, sepsis-associated complications, such as acute kidney injury, acute respiratory distress syndrome, sepsis-induced myocardial dysfunction, hepatic dysfunction, brain dysfunction, and skeletal muscle wasting, significantly contribute to the systemic biochemical profile (Ning et al., 2021). Indeed, the cytokine-driven inflammatory dysregulation (such as elevations in IL-6 and IL-1β) is not only a marker of sepsis but also a core driver of ARDS pathogenesis and progression (Zhou and Lu, 2024). Furthermore, the crosstalk between systemic inflammation and organ-specific barriers, such as endothelial dysfunction and impaired myocardial energy metabolism, further amplifies the elevation of pro-inflammatory factors like IL-6 and TNF-α (Vella et al., 2025). These comorbidities can cause changes in various biomarkers, affecting the accuracy of diagnosis and prognosis assessment of sepsis. IL-6, belonging to the IL family, is immediately and transiently released in response to infections and tissue injuries and evokes host defense by stimulating acute phase reactions, hematopoiesis, and immune responses (Tanaka et al., 2014). In a prospective controlled study, IL-6 has shown a superior diagnostic and prognostic performance for sepsis and septic shock to pentraxin 3 and PCT (Song et al., 2019). Another comparative study has reported that IL6 has better kinetics for monitoring the therapeutic efficacy of antibiotics (Jekarl et al., 2013). These studies all contributed to a good diagnostic and prognostic performance of IL-6 for septic patients, which was also demonstrated in our study. Deniz et al. found elevated NLR and APACHE II scores in the deceased patients relative to the survived patients in the ICU (Deniz et al., 2022). Another recent study also demonstrates a positive correlation between NLR and APACHE II scores in bloodstream infection and sepsis patients, and it indicates PCT, NLR, and APACHE II scores to be independent hazardous markers for 28-day mortality (Liang and Yu, 2022). Additionally, NLR shares a positive correlation with the severity and prognostic scores of sepsis on admission (Velissaris et al., 2018). Herein, our data based on ROC curves and multivariate logistic regression exhibited consistent presentation with the diagnostic and prognostic significance mentioned in these studies.

More importantly, our study proposed that combined detection of IL-6, NLR, and APACHE II scores had higher AUC, sensitivity, and specificity than any one of them, showing a better prognostic performance than a single indicator. A combination of any two biomarkers (CRP, PCT, and IL-6) shows favorable performance in predicting the hyperinflammatory state as well as increased diagnostic specificity for pediatric sepsis (Zeng et al., 2022). The combined detection of WBC, serum PCT, IL-6, and CRP levels also exhibits a superior diagnostic potential for neonatal sepsis to the single detection (An et al., 2023). A recent study has suggested that detection of NLR together with IL-6 can remarkably improve the predictive value of 28-day mortality (Liu et al., 2021). A combination of monocyte/high-density lipoprotein cholesterol ratio with NLR seems to improve the predictive efficacy for the risk of 28-day mortality in septic patients (Li et al., 2021). In our research, the combined detection of IL-6, NLR, and APACHE II scores exhibited high predictive value for 28-day in-hospital mortality in septic patients. These three factors were identified to be independent risk indicators for 28-day in-hospital mortality, and higher levels of serum IL-6, NLR, and APACHE II scores were linked to raised risks of 28-day in-hospital mortality in septic patients.

## Limitations

IL-6 is a pro-inflammatory cytokine whose elevation is a direct consequence of the innate immune response. Pathogens (such as bacteria and viruses) trigger the release of pathogen-associated molecular patterns (PAMPs), which, along with damage-associated molecular patterns (DAMPs), are recognized by Toll-like receptors (TLRs). This cascade activates immune cells to produce an inflammatory escalation, leading to multi-organ injury (Moriyama and Nishida, 2021; Alcala et al., 2022). Similarly, an elevated NLR serves as a robust indicator of the cellular immune imbalance, particularly in bacterial sepsis. Bacterial toxins and cytokines stimulate massive release of neutrophils while simultaneously inducing lymphocyte apoptosis and suppressing their proliferation. This leads to a significantly increased NLR (Benschop et al., 1996; Dragoescu et al., 2021). Furthermore, sepsis patients often present with comorbidities that may confound biomarker levels, necessitating integrated clinical assessment for accurate diagnosis. Antibiotic therapy is typically initiated immediately upon sepsis diagnosis. Such antibiotic intervention rapidly reduces pathogen load, thereby suppressing the synthesis and release of inflammatory cytokines such as IL-6, as well as lowering PCT levels (Formenti et al., 2024; Nandi et al., 2025). Moreover, septic or septic shock patients receiving corticosteroid therapy (such as hydrocortisone) experience a downregulation of pro-inflammatory responses, while the innate immunity is preserved and the anti-inflammatory response is limited (Marik, 2018). This may suppress the levels of inflammatory cytokines like IL−6 and CRP while affecting neutrophil activation, potentially causing the NLR to deviate from its true pathophysiological level (Ma et al., 2024). However, the data in this study were collected only at the initial admission stage without post−treatment dynamic monitoring. Consequently, early interventions with antibiotics or steroids could have altered the baseline levels of inflammatory markers (such as IL−6 and NLR), which may undermine the accuracy of analyses examining the association between these indicators and sepsis severity. Finally, as a single−center retrospective study, the present study is susceptible to selection bias due to its limited sample size. Generalizability of the findings may also be constrained by the specific characteristics of single−center data and the inherent limitations of a retrospective design, thereby restricting the broader applicability of the conclusions.

## Conclusion

This study indicates that combined detection of IL-6, NLR, and the APACHE II score improves the predictive performance for septic shock and 28−day in−hospital mortality, with both sensitivity and specificity exceeding 75%. Moreover, elevated levels of these three markers were closely linked with an increased risk of 28−day in−hospital mortality in septic patients. These findings provide a theoretical foundation for developing more reliable prognostic models for sepsis and offer new perspectives for personalized management, risk stratification, and early intervention in sepsis. In the future, we will integrate multi-dimensional biomarkers (e.g., inflammatory cytokines, metabolites) and omics technologies to further optimize the diagnosis, stratified management, and prognostic management of sepsis.

## Data Availability

All data generated or analysed during this study are included in this article. Further enquiries can be directed to the corresponding author.
