# Supplementary figures and images for: Severity and 28-day in-hospital mortality of interleukin-6, neutrophil to lymphocyte ratio, and APACHE II score in patients with sepsis
Source: Front Cell Infect Microbiol. 2026 Mar 25;16:1690126. doi: 10.3389/fcimb.2026.1690126 (PMC13057303; doi:10.3389/fcimb.2026.1690126)

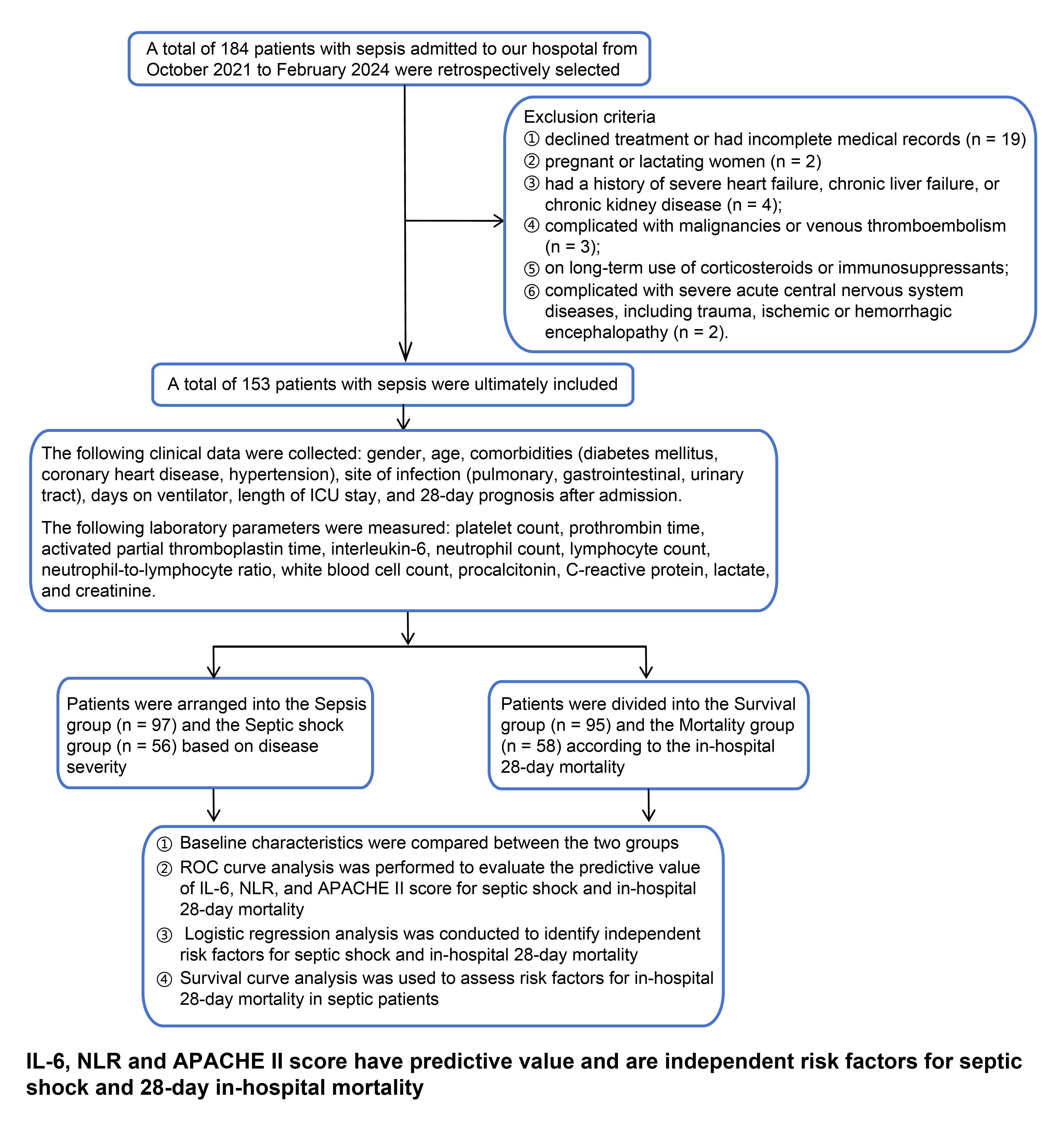

Supplement: Supplementary file 1 [file Image1.tif]
